# Foreign

**Published:** 1842-02-19

**Authors:** 


### FOREIGN.

*On the Preparations of Extractum Colchici Acetum and the Acetum Cantharidis.* By Sir CHARLES SCUDAMORE.—In the 4th edition of my Treatise on the Nature and Cure of Gout and Gravel, &c. published in 1823, I expressed myself as follows, on the subject of the acetic extract:—"For the last two or three years, I have been in the habit of prescribing an extract obtained from the acetum colchici by inspissation, which, in order to secure uniformity in the medicine, I have hitherto directed as prepared at my desire, by Mr. Gordon, of Oxford Street. I have never in any instance found it disagree in any respect, and I consider it a very useful and convenient form of medicine."

The method in question, to which I give the preference, consists simply in inspissating, by means of a gentle heat, a saturated infusion of the dried roots (carefully selected) of the colchicum autumnale in acetum destillatum; the evaporation being carried on till the mass became of the consistency of honey. I am convinced that this is a superior preparation to that of the Pharmacopœia, more active in its properties, and more uniform in its strength. It seems reasonable to believe, that the root in the dried state is in a more favourable condition for yielding its active principles than when fresh. I am of opinion that with these the acetic acid forms such combination as to furnish a medicine of superior properties; and I am strengthened in my partiality by the increased favour which it daily finds with the profession. From the best estimate which we can make, we are of opinion that one grain of this extract represents a fluid drachm of the acetum colchici. Yet it may certainly be given with more freedom than the fluid preparation, as not tending to disorder the stomach, and allowing so conveniently other medicines to be given in conjunction with it, calculated to favour further indications than that of counteracting the gouty or the rheumatic irritation, the only legitimate purpose to be answered by this medicine when prescribed for these disorders. In my view of the question, as I have often had occasion to observe in my different publications, colchicum, in no form in which it may be employed in the treatment of gout, should be considered in any other light than as a palliative remedy; and I think the greatest error committed in its administration has been the trusting it as a curative agent; neglecting the more important consideration of looking to the exact state of the constitution, and ascertaining the visceral conditions or other circumstances, in relation to which the gout may stand but as a symptom. Consequently, also, no two cases will allow of being treated exactly alike; and hence the folly, too, of the empirical conduct of patients themselves. I affirm of colchicum that it is the best or the worst remedy in gout, accordingly as it may be employed.

There is less objection, I conceive, in principle to the separate administration of colchicum in rheumatism than in gout, as the disease may not necessarily be so much connected with, and depending upon, internal visceral disorder; but here, also, a combined treatment will be found the most rational and useful.

In the 2d edition of my "Cases illustrating and confirming the Remedial Power of the Inhalation of Iodine and Conium in Tubercular Phthisis, and various disordered states of the Lungs and Air-Passages," I gave the following statement of the other preparation of which I am about to speak,—the acetum cantharidis:—"It happens with some persons that even a blister causes a degree of irritation too disturbing for the general system. I have used with advantage as a convenient counter-irritant a saturated infusion of cantharides in the strongest acetic acid. It is a very manageable remedy, and in many ways highly convenient. If used diluted, it will act as a rubefacient; if in its state of concentration, it will vesicate in a short time. It may be applied by means of a strong camel's-hair brush to the smallest extent of surface, and in any situation.

On other occasions I have, in the journals, adverted to this remedy. I do not recommend it as one calculated to supersede the use of the ordinary blister plaster, which I consider to have the advantage of acting more on the deeper seated vessels, and in its vesication producing a more dense serum, and by a more gradual operation, which is often desirable. When, however, a very prompt counter-irritation and vesication may be desired, and also when particular situation or other motives of convenience come to be considered, this liquid blister is a very valuable resource.

The mode of preparing it, as I have mentioned, is very preferable to the formula directed in the Pharmacopœia. The acetic acid in its state of concentration must be a more powerful solvent of the active principles of the cantharides than the common acetic acid, as well as being in itself very efficient in producing vesication.—*London Med. Gaz.* December 10, 1841.

---

*Most Obstinate Hiccup cured with Quinine.*—A countryman was seized, while recovering from an attack of ague, with a convulsive hiccup, which recurred every few moments. A variety of antispasmodics; such as ether, valerian, musk, assafœtida, opium, &c. were tried, but without relieving the troublesome symptom; blisters also, as well as other cutaneous irritants, were applied with equal unsuccess. For nine days the hiccup continued with but little intermission, and this was only at irregular intervals for about a quarter of an hour at most.

Suspecting that, although all the other symptoms of the ague had ceased for some time, this convulsive affection of the stomach might somehow be connected with it, the medical attendant administered a large dose of quinine in an enema. A few hours after it was given, the hiccup ceased almost entirely; but it again returned, as violent as before, next day. The quinine was again ordered, and with equally happy effects; and, by persevering in its use for a few days, the symptoms did not return.—*Med. Chir. Rev., from Journal des Connois. Med. Chir.*

---

*Walnut-leaf Tea, a good remedy in Scrofulous Complaints.*—Professor Negrier, of Angers, a respectable authority, has for some years past been trying the effects of an infusion of the leaves of the walnut tree in a variety of scrofulous maladies, and the results of his experience have led him to form a most favourable opinion of it as an antiscrofulous remedy.

He reports a great number of cases of disease of the lymphatics, with or without ulceration of the integuments, of scrofulous ophthalmia, of affections of the bones and periosteum, &c. in which decided and very marked benefit was obtained from a course of this simply prepared tea. A handful of the fresh or slightly dried leaves may be added to a pint of boiling water, and of this infusion a small cupful may be taken twice a day. An extract may also be prepared by evaporation, and this Dr. Negrier recommends to be given at the same time, either in the form of pills or of a thick syrup. A strong decoction of the leaves he has used with excellent effects as an application to scrofulous ulcers.—*Ibid., from Archives Generales.*

*Remark.*—We know nothing practically of this remedy, but somehow or other we feel inclined to predict that it is one deserving notice, and that it will be found useful in some cases of lymphatic and cutaneous disease. Whenever we pass a walnut tree, and smell the peculiar odour which it gives out, the idea always occurs to us that it is not destitute of tolerably active medicinal properties. Moreover, as a general remark, scrofulous diseases are, on the whole, much more benefited by vegetable remedies than by those of a metallic nature, except, perhaps, steel; and this we know is more *kin*, so to speak, to the body than any other of its class.—(*Rev.*)

---

*Homœopathy Exposed.*—The papers have been circulating the following paragraph.

"The Duke of Canizzaro died from taking three pills at once, ordered to be taken singly, either through his own mistake, or through that of his *homœopathic* physician, and that these pills contained *ar-*

*senic.* Thus we see a nobleman, in the enjoyment of a large fortune, dying, *poisoned like a rat.* Considering these pills were prescribed in conformity to *homœopathic* practice, in which only *millionth* doses are supposed to be used, so that a few hundred thousand portions might be taken without producing death, one can but look upon this result as no less extraordinary than unfortunate. It gives rise to no little matter of reflection upon the source of the active effect of these doses of fabulous diminutiveness, and it shows that those optimists may err who think that homœopathy is a mere hocus-pocus, like the *papato* of the seventeenth century."

We have always thought and said that the clever rogues among the homœopathists take good care to give active doses of medicine under cover of their infinitesimal humbug. Here is a case in point. How could the Duke of Canizzaro die from swallowing two or three hundred millionths of a grain of arsenic? The quackery and imposture of the thing are palpable. But it is of no use telling the public to avoid quacks. They *will* be gulled, and therefore individuals, like the Duke of Canizzaro, must pay for it.—*Ibid.*

---

*On Rheumatic Dermalgia, or Rheumatism of the Skin.* By J. H. S. Beau, Physician to the Central Bureau of the Hospitals of Paris.—Neuralgia of the skin has hitherto been usually confounded with pains of the nervous trunks, muscles, &c. M. Piorry was the first who referred it to a separate head under the name of *dermalgia.* It frequently coexists with neuralgia of the nervous trunks, with ramollissement of the brain; or occurs in cases of inflammation of the spinal cord. Severe pain in the uterus is often attended with dermalgia of the skin of the pelvis and thighs, and clavus hystericus is frequently a neuralgic affection of the skin.

There are several other forms of this affection, but one which has escaped notice down to the present time is rheumatic dermalgia. This is of more frequent occurrence among men than women, and is induced by damp, cold, and those other causes to which rheumatism generally is owing. Hence it is most common at the beginning of spring. The head and lower extremities are the parts usually attacked, but the pain is not stationary in one place; often changing its seat in a gradual manner, just as erysipelas sometimes wanders from place to to place. Patients experience two kinds of pain, the one abiding, the other intermittent and severe, resembling the prick of a pin or an electric shock, and recurring about every thirty seconds. The abiding pain is frequently little more than a permanent exaltation of the natural sensibility of the skin. Friction of the part with the finger or with the patient's dress, always increases the pain; and if the affected part is covered with hair, very severe suffering may be produced by passing the hand over the hair. The intermittent pain is often at once excited by touching the part in this manner, and though firmer pressure puts a stop to the permanent pain, the return of the intermittent pain cannot be thus prevented. The intermittent pain is always considerably worse at night. Rheumatism of the skin usually alternates with that form of the disease which affects the muscular and fibrous tissues. Its usual duration is from a day to a couple of days, and it subsides by degrees just in the same way as it made its attack. The author met with three instances in which it was accompanied with fever and involved a much larger surface of skin than usual. It is, in general, an affection easily curable. The indications for its treatment do not differ from those to be observed in ordinary rheumatism, but it does not generally require any very active remedial measures. To prevent its recurrence, it is always desirable for the patient to wear flannel next his skin.—*Brit. and For. Med. Rev.*, from *Arch. Gen. de Med., Septembre,* 1841.

---

*Account of an Epidemic of Trismus Neonatorum, which occurred in the Lying-in Hospital at Stockholm in the year* 1834. By P. G. Cederschjöld.—Before the outbreak of the disease, sporadic cases of convulsive affections had been met with among the infants, putting on sometimes the form of trismus or tetanus, at other times differing but little from ordinary convulsions.

The commencement of the epidemic may be referred to the month of February, 1834, when one child was attacked by the disease. Three cases occurred in March, and two in April; but from the 8th of April to the 24th of May, the disease did not again show itself. From the 24th of May, however, to the 23d of June, 16 children were attacked by trismus, of whom 9 died. Another interval of 10 days elapsed without any fresh cases appearing; but between the 3d and 14th of July, three children died of trismus, and between the 14th and 21st, nine more infants were attacked and five died. After this time the cases of the disease became much less frequent, though it did not finally disappear until the 15th of November.

During the continuance of the epidemic, 42 children were attacked by the disease, of whom 34 died, but it is probable that some of the cases reported as cured were not in reality instances of the disease, since no child recovered in whom the symptoms of trismus were at all well marked. Professor Cederschjöld attributes the prevalence of the disease epidemically during the months of May, June, and July, to the great variableness of the weather; but the circumstance of its not having occurred among the children of women delivered at their own homes, renders it probable that it was owing to some local cause.

The first symptoms usually appeared between the 4th and 6th day after birth, and the duration of the disease seldom exceeded two days. When attacked, a child became uneasy, cried aloud sometimes, and did not suck, though it was eager to take the breast. A slight convulsive movement was next observed about the lips, drawing apart the angles of the mouth. A convulsive seizure would next follow, sometimes resembling an ordinary convulsion, but at other times attended with trismus or tetanus. The alternation of trismus with general convulsions was frequently observed, but tetanus and convulsions were not nearly so often combined. Three periods might be observed in the convulsive attacks: the first was attended by symptoms of suffocation; during its continuance the whole body was stiff, and the face livid; convulsive movements, especially of the face and eyes, succeeded, and the livor disappeared; the third stage then came on, during which the child foamed at the mouth, and breathed with difficulty and a snoring sound. The attacks of tetanus occurred at longer intervals than those of convulsions; they were attended with opisthotonos, but seemed to exhaust the infants less than the convulsions.

Great congestion of the membranes and substance of the brain and spinal cord were found after death, and sometimes an extravasation of coagulated blood at the base of the cranium. The lungs were much collapsed, and often appeared to have been only incompletely inflated. The liver was usually much congested, and the gall-bladder distended.

The treatment consisted in the application of leeches to the nape of the neck, the administration of nauseating doses of emetics, and the use of antispasmodics and opium. Mild purgatives followed by antispasmodics seemed to be beneficial in some cases where premonitory symptoms of the disease had appeared; and laxatives, with the separation of the sick from the healthy, were employed as prophylactic measures.—*Brit. and. For. Med. Rev.*, from *Neue Zeits. fur Geburtskunde.* Band x. Heft iii. 1841.

---

*Observations on the management of the Placenta.* By Edward W. Murphy, M. D., late Assistant Physician to the Dublin Lying-in Hospital.

[The following extracts from a somewhat unnecessarily prolix paper in the London Medical Gazette for Nov. 12, 1841, are deemed worthy the attention of our readers, as displaying a beautiful practical application of a principle for which we have always contended, namely, that due tonic contraction of the parieties of a cavity is essential to the vigorous exercise of the functions of its within contained organs, as due tonicity of the skin and free cellular tissue is necessary to the proper display of nervous or vascular functions of the surface.

*Temporary and moderate mechanical support* is indicated in all cases of such lack of tone, occurring in parts capable of recovering their energies by rest.]

Denman states, "that if the placenta be not expelled at the end of *four hours* from the birth of the child, it is generally wise to determine upon extracting it." Since his time, this period has been limited to two hours, one, and even half an hour. Dr. John Clarke, by a series of careful observations, determined that the ordinary period in which the uterine pains return is from 20 to 25 minutes, when the placenta should be expelled at least into the vagina; but as it might be suffered to remain a longer time without danger, each practitioner adopted a different rule within these limits.

Before, however, proceeding to introduce the hand into the uterus, different means were put in practice to secure its removal. If the placenta was ascertained to be in the vagina by the finger reaching the insertion of the funis, the hand was passed partially up, in the manner directed by Smellie, and the placenta withdrawn: if within the womb, which was assumed to be inert, attempts were made by frictions over the abdominal covering of the uterus to excite its action. Dewees observes, "All attempts to deliver the placenta must be forborne until we have, by properly instituted frictions over the region of the uterus, obliged it to contract and harden itself under the hand, and, at the same time, retire lower into the pelvis," (p. 513.) Burns recommends "pressing on the uterine region, and rubbing the abdominal covering, or gently grasping the womb through the relaxed parietes." In the same way even the latest writers of the present day might be quoted to prove that the general practice is, after the child is born, to leave the womb for a certain time to itself to expel the placenta, which, if it does not take place, *inertia uteri* is assumed, and means adopted to excite its action, failing in which the hand is introduced. The abdominal bandage was never applied by Denman before the sixth day after delivery, who very naturally shuddered at its abuse, when it was applied for the purpose of squeezing the patient into shape. It was considered by Dr. H. Ley as useless, and therefore objected to; and, of the present day, Drs. Collins and Conquest are the only British writers I have met with who recommend its application previous to the expulsion of the placenta. In all this varying treatment, the influence produced on the uterus by its altered relation to the abdominal parietes seems to have attracted but little notice, nor has the agency of the muscles of the abdomen in stimulating the action of the uterus received the attention it merits.

It is necessary to consider the condition of the uterus previously and subsequent to the expulsion of the child: We find every expulsive effort of the mother during the passage of the child engaging the diaphragm and abdominal muscles in powerful action; an equable pressure is thus conveyed over every part of the fundus uteri, which must essentially contribute to maintain its action. As soon as the child is expelled, if it were in a state of nature, or with a healthy strong woman, the abdominal muscles contracting on the contents of the abdomen, continue a uniform pressure on the retiring uterus, and therefore, if the placenta be not detached on the delivering of the child, the uterus is not permitted to relax, but, with the returning pain, is compressed on all sides by the abdominal viscera; being thus stimulated to increased action, the placenta is expelled gradually and safely by successive contractions. Such would be the process of nature in a healthy and vigorous constitution, where the walls of the abdomen retain their strength and elasticity; but while we thus look at the powers of nature to empty the uterus of its contents, it is necessary to recollect the influence of our civilized habits in interfering with its exercise : an atonic condition of the muscles of the abdomen would lead to very opposite results : the uterus, deprived of the support it should have received, would cease to act as soon as it ceased to be excited by the limbs of the child. The placenta within the uterine cavity would at first excite a few feeble pains until the uterus became accustomed to its presence ; when these efforts, not being supported, would cease, and the placenta be retained by what is called inertia uteri. The atony existing, however, much more in the abdominal covering than in the uterus, as soon as means are adopted to supply what these muscles would naturally accomplish, and that the fundus of the uterus is compressed by the hand, it is no longer inert, but immediately obeys the stimulus, and expels the placenta. Such are precisely the circumstances under which retentions of the placenta frequently occur. After the birth of the child, the uterus remains for some time in a state of contraction, as may be observed by placing the hand on the fundus. After an interval varying from five to fifteen minutes (the uterus generally continuing in this contracted state,) a slight pain will return, but the uterus, not being supported, is much more relaxed after the pain than before it. If the funis be drawn, the uterus may be again excited, and a similar pain return ; but being insufficient for the purpose, the placenta is not detached, the practitioner ceases from any further attempts to remove it by such means, and determines, as no hæmorrhage has taken place, to wait for whatever period he has determined in his own mind would authorize him to introduce his hand into the uterus; then, having previously adopted the means already stated, frictions, &c., he proceeds to its removal, if they fail. In such a case, the placenta is generally found lying in the uterus, detached, and the case is set down as inertia uteri. If, however, the attendant be not so patient, but will draw the funis, frequently pass his fingers up to feel the insertion, and so irritate the lower section of the uterus, the chances are that it will be thrown into a state of irregular contraction, rendering its removal much more difficult, and be described as hour-glass contraction of the uterus.

That atony of the abdominal muscles, rather than inertia uteri, is the cause of such retentions, may readily be admitted. From the practice of compressing the abdomen to acquire a fashionable but most unnatural shape, its muscles become comparatively inert even before pregnancy takes place : the influence of this atonic condition upon the contents of the abdomen, the weakened peristaltic action of the intestines, their coats readily yielding to flatus, and sometimes even distended to the utmost from the same cause when increased by disease, are facts obvious to the experience of every physician. It is not surprising, therefore, that when a weak muscle is over distended by the gravid uterus, it should be unable to contract itself sufficiently when the uterus recedes from it, and that consequently the latter, deprived of the support it should receive, ceases to act, and retains the placenta. If, on the other hand, such support be artificially supplied, by a bandage firmly and equably put on, or by the hands steadily compressing the fundus uteri, the risk of such retention may not only be prevented, but in cases where it has already taken place the uterus may be made to act with sufficient vigour to throw off the placenta without further trouble. For further evidence in favour of this view, I may refer to the late Dr. Joseph Clarke, of Dublin, who had early observed the importance of this principle since adopted by Dr. Collins and others, viz. "by pursuing, with a hand on the abdomen, the fundus uteri in its contractions until the fœtus be entirely expelled, and afterwards, continuing for some time this pressure, to keep it, if possible, in a contracted state."—*Collins*, 121. "Labours thus conducted," says Dr. Clarke, "will be less likely to be followed by retention of the placenta, uterine hæmorrhage, or after-pains."

[After some remarks on the former terrible abuses of bandaging during and after labour, and the contrary purposes and opinions of modern writers, Dr. Murphy proceeds.]

"As an effectual agent in accomplishing what the abdominal muscles too often fail to do, to preserve an equable pressure over the fundus uteri, and to secure its uniform contraction, I have found a properly applied bandage of the greatest service in assisting the uterine pains; but, in order to produce the effect, it is necessary to recollect the intention. A bandage should not be applied merely for the purpose of binding the pelvis, or of constricting the abdomen over the fundus uteri—a method of application which becomes, sometimes, intolerable to the patient: it should include the whole of the abdomen, being drawn with the greatest tightness around the pelvis, and gradually less so as we approach the diaphragm. The practice of using a narrow bandage, that will hardly reach above the fundus uteri, seems to me worse than useless. To the patient it produces all the unpleasant constriction of a belt tightly drawn round the loins; while the uterus is compressed by the bandage alone, and the intestines, being left free in the upper part of the abdomen, project it over the binder, causing a very unsightly appearance, and depriving the posterior and lateral portions of the uterus of the pressure which should be conveyed, through them, to these parts. The application of a bandage, under the circumstances herein stated, must not be confounded with its use in cases of true inertia uteri, in which there is severe hæmorrhage and very great difficulty in maintaining its permanent contraction. In such instances the most powerful compression, by means of graduated pads, and a firmly applied binder, is often necessary to prevent relaxation and a return of the hæmorrhage."

---

*The Medical Examiner* is published every Saturday by *J. C. Turnpenny*, N. E. corner of Spruce and Tenth streets. Terms—*three dollars* a year for one copy, or *five dollars* for two copies sent to the same post office, in all cases in advance. Postage the same as on newspapers. Letters to be addressed,—post-paid,—to the Editors of the Medical Examiner, Philadelphia.
*Reynell Coates, M. D.*, Acting Editor.
*J. B. Biddle, M. D.*, and *W. W. Gerhard, M. D.*, Co-editors.
*J. B. Biddle, M. D.*, Proprietor.

